# The Role of Energy Availability in Reproductive Function in the Female Athlete Triad and Extension of its Effects to Men: An Initial Working Model of a Similar Syndrome in Male Athletes

**DOI:** 10.1007/s40279-019-01217-3

**Published:** 2019-11-06

**Authors:** Mary Jane De Souza, Kristen J. Koltun, Nancy I. Williams

**Affiliations:** Women’s Health and Exercise Laboratory, Department of Kinesiology, The Pennsylvania State University, University Park, PA 16802 USA

## Abstract

The Female Athlete Triad represents three interrelated conditions of (i) low energy availability (energy deficiency), presenting with or without disordered eating, (ii) menstrual dysfunction, and (iii) poor bone health, each of which can exist along a continuum of severity ranging from mild and moderate subclinical health concerns to severe clinical outcomes, including eating disorders, amenorrhea, and osteoporosis. This review provides a brief overview of the Female Athlete Triad, including updating the current thinking regarding energy availability and how it relates to reproductive function, and sets the stage for an initial working model of a similar syndrome in males that will be based on currently available evidence and will later be defined and referred to as a Male Athlete Triad by the newly re-named Female and Male Athlete Triad Coalition. A primary focus of this paper will be on the physiology of each Triad model with an emphasis on low energy availability and its role in reproductive function, with a brief introduction on its effects on bone health in men. From the data reviewed, (i) a specific threshold of energy availability below which menstrual disturbances are induced is not supported; (ii) it appears that the energetic, reproductive, and bone systems in men are more resilient to the effects of low energy availability compared to those of women, requiring more severe energetic perturbations before alterations are observed; and (iii) it appears that recovery of the hypothalamic pituitary gonadal axis can be observed more quickly in men than in women.

## Key Points

**Table:** 

An absolute energy availability threshold of 30 kcal/kg fat free mass/day, below which menstrual disturbances are induced in exercising women, is not supported.
Energy availability should be used in conjunction with other measurements of metabolic status and eating behaviors to assess an at-risk profile for Triad sequelae, such as body weight and composition, resting metabolic rate, and measures of restrictive or under-eating.
There is evidence for a similar Triad-like syndrome in men where energy deficiency/low energy availability impacts reproductive function and bone health; however, men appear to require more severe energy deficits than women for an impact to be observed.

## Introduction

The Female Athlete Triad model, originally presented in 1997 [1], and updated in 2007 [2], represents the scientific underpinnings and clinical sequelae associated with (i) low energy availability (energy deficiency), presenting with or without disordered eating, (ii) menstrual dysfunction, and (iii) poor bone health. Exercising women, including competitive and non-competitive athletes and those who are recreationally active, can be impacted by the Triad along a continuum of severity ranging from mild and moderate subclinical health concerns to severe clinical outcomes, which includes eating disorders, amenorrhea, and osteoporosis (with or without fractures) [2].

The past three to four decades have been a period of much research engagement that has drastically improved our understanding of the primary components of the Female Athlete Triad. The research to date has provided an evidenced-based model with clear mechanistic descriptions of how each component of the Triad interacts with each of the other components, and the Triad model has been developed and defined from scientifically rigorous research methodologies [3]. This research has been translated in the form of treatment and return-to-play guidelines for the Female Athlete Triad and its associated medical conditions, which were developed by the Female Athlete Triad Coalition in 2014 [4, 5]. The American College of Sports Medicine has convened a consensus group to update the Female Athlete Triad in 2019.

The Female and Male Athlete Triad Coalition will publish its first consensus statement on the Male Athlete Triad late in 2019. This paper will represent the first official consensus statement on the Male Athlete Triad. Although several papers have addressed Triad-like issues in male athletes [6–8], the consensus statement will represent formal action to highlight the important effects of exercise in male athletes and will offer strategies for prevention and recommendations for management.

**Fig. 1:**
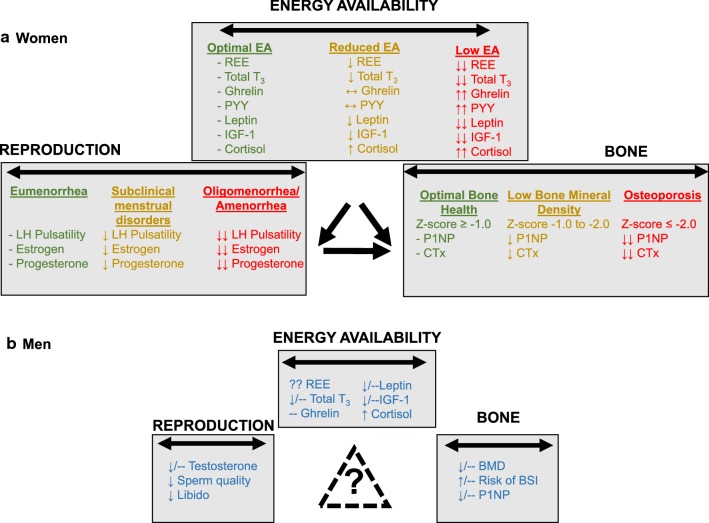
Comparison of evidence available to date regarding the effects of low energy availability on reproduction and bone health in exercising men. **a** The Female Athlete Triad represents a spectrum of energy availability, menstrual health, and bone health status. Within each health category, individuals may vary from optimal health (denoted in green) to compromised health (orange) to a pathological health status (red). **b** Summary of effects of low energy availability on metabolism, reproduction, and bone health in men supporting the possibility that a similar Triad-like condition may exist in exercising men. *REE* resting energy expenditure, *T*_*3*_ triiodothyronine, *PYY* peptide YY, *IGF*-*1* insulin-like growth factor 1, *LH* luteinizing hormone, *P1NP* N-terminal propeptide of type I collagen, *CTx* C-terminal telopeptide type I collagen, *BMD* bone mineral density, *BSI* bone stress injury, – indicates no change, ↓ indicates reduced, ↑ indicates increased, ?? indicates unknown

This review provides a brief overview of the Female Athlete Triad, including an update on the current thinking around an energy availability threshold as it relates to reproductive function, and sets the stage for an initial working model of a similar Triad syndrome in male athletes based on currently available evidence. A primary focus will be on the physiology of each Triad model with an emphasis on low energy availability and its role in reproductive function, as presented in Fig. 1. This paper does not address treatment guidelines nor recommendations for return to play since such recommendations are available elsewhere for female athletes [4, 5], and a detailed paper on both non-pharmacological (nutritional) and pharmacological therapy for the Female Athlete Triad is also available elsewhere [9]. As evidence accumulates, treatment and return-to-play guidelines will be developed for male athletes by the Female and Male Athlete Triad Coalition.

## Review and Update on Energy Availability Associated with the Female Athlete Triad

### The Model

The Female Athlete Triad model, as updated in 2007 [2], presents each component of the Triad along a bi-directional continuum of severity, ranging from “healthy” to “unhealthy,” and includes an “intermediate” subclinical midpoint. The “unhealthy” end of the continuum represents the most severe clinical endpoints and includes low energy availability (with or without disordered eating), amenorrhea, and osteoporosis. At the “healthy” end of the continuum, health conditions are presumed to be optimized, i.e., there is adequate energy availability such that energy intake matches energy expenditure needs, ovulatory menstrual cycles are maintained, and bone health is generally good with the absence of stress reactions or fractures [2]. The “intermediate” points along the continuum represent subclinical manifestations of the severe clinical endpoints and include menstrual disorders such as luteal phase defects and anovulation, and unfavorable changes in bone health. The nature of this 2007 continuum model underscores the unique presentations of affected athletes and exercising women such that each individual may progress or recover along the three continuums at varying rates, and by the nature of the bi-directional arrows, move in a direction toward progression of the Triad or recovery from the Triad [2]. The direction of the arrows that link each of the three continuums indicates, in a scientifically rigorous, evidenced-based manner, how each component of the Triad is related to another component. For example, unidirectional arrows from low energy availability to menstrual dysfunction and poor bone health and from menstrual dysfunction to poor bone health represent the causal roles of (i) low energy availability on menstrual disorders and on poor bone health, and (ii) menstrual disorders and hypoestrogenemia on poor bone health. The Triad model also incorporates disordered eating behaviors by indicating that low energy availability can occur with or without the presence of disordered eating, and may also include eating disorders [2].

### Low Energy Availability in the Etiology of the Triad

The most clinically significant symptoms of low energy availability in female athletes and exercising women, and therefore the foundation of the Triad model, are its effects on the menstrual cycle and on bone health. Although other symptoms are apparent, such as endothelial dysfunction [10], altered lipid profiles [11], and, in one case, poor sport performance [12], the physiological impact of low energy availability on the menstrual cycle and bone are most clinically significant since they both render negative health effects. In mammals, available oxidizable fuel is partitioned into compartments important for viable function and sustenance, including thermoregulation, cellular maintenance, locomotion, growth, immune function, and reproductive function [13, 14]. During times of limited fuel availability, energy is shunted or re-partitioned away from growth and reproduction in order to prioritize the compartments vital for survival of the individual, such as thermoregulation, cellular maintenance, and locomotion [13, 14]. As such, available energy is shunted away from growth and reproduction, resulting in a cascade of metabolic and energetic alterations that conserve energy. Energy expenditure is thus conserved through suppression of resting metabolic rate, total triiodothyronine (TT_3_), insulin-like growth factor-1 (IGF-1), leptin, and insulin, and counter regulatory upregulation of cortisol, and the development of an acquired growth hormone (GH) resistance [4, 5]. These metabolic and endocrine adaptations occur within normal physiological ranges and represent expected adjustments to low energy availability.

The causal role of low energy availability/energy deficiency in perturbations to energetic homeostasis and reproductive function was defined by the (i) careful, short-term (4–5 days) experimental manipulations of varying levels of energy availability on energetic hormones and luteinizing hormone (LH) pulsatility conducted by Dr. Anne Loucks [15, 16], and (ii) randomized controlled trials (two to three menstrual cycles) conducted by Drs. Bullen [17, 18] and Williams [19, 20] on the actual induction of menstrual disturbances. One of the most important findings of these experiments is that even mild to modest reductions in energy availability, in both the short-term model of Loucks et al. [15, 16] and the long-term models of Bullen et al. [17, 18] and Williams et al. in both monkeys [21] and women [19, 20], resulted in significant suppression of metabolic hormones, suppression of LH pulse frequency, and the induction of abnormal menstrual cycles, including the suppression of ovulation and estradiol concentrations without significant reductions in body weight. These findings, however, stand in stark contrast to what has been observed in men, in whom far greater reductions in energy availability/energy deficiency appear necessary to impart similar effects on metabolic hormones and testosterone. Indeed, in two different short-term experiments lasting 4–5 days each, energy availability was manipulated in men to a level considered “severely low” (15 kcal/kg fat free mass [FFM]/day) based on the experiments completed in women. In contrast to women, this level of energy availability resulted in few metabolic alterations and did not alter serum testosterone [22, 23]. The details of these data in men are discussed in the next section.

### A Threshold of Low Energy Availability and Relationship to Reproductive Sequelae

Our understanding of a threshold of energy availability, below which LH pulsatility was disrupted and metabolic hormones were suppressed, was established by the short-term experiments of Dr. Loucks [15, 16]. Loucks et al. demonstrated that LH pulsatility patterns were disrupted when energy availability was below 30 kcal/kg FFM/day [15], a threshold deemed to be equivalent to that required to maintain resting metabolic rate. Below this threshold of 30 kcal/kg FFM/day, it was proposed that menstrual disturbances would be apparent; however, only LH pulsatility, a surrogate marker of menstrual function, was assessed in these short experiments, not actual menstrual cycle changes.

**Fig. 2:**
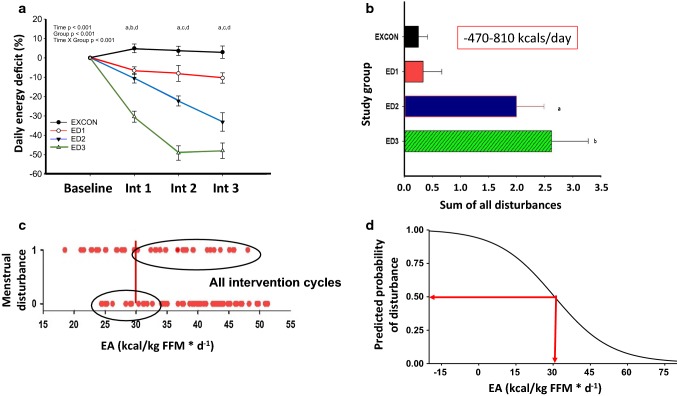
**a** Average daily energy deficit (%) experienced across study phases and,  **b** sum of all menstrual disturbances across the intervention for each group. Data are reported as mean ± SE. **a** ^a^ED1 significantly different from EXCON; ^b^ED2 significantly different from EXCON; ^c^ED2 significantly different from EXCON and ED1; ^d^ ED3 significantly different from EXCON, ED1, and ED2. **b**^a^significantly different from EXCON; ^b^significantly different from EXCON and ED1. Reproduced from Williams et al. [19], with permission. **c** Individual EA data and the incidence of MD during intervention and all intervention cycles combined. *Y* axes notations are 0, no MD indicating an ovulatory cycle; 1, at least one MD indicating a luteal phase disturbance, oligomenorrhea, or anovulation; each dot represents one participant’s average EA and whether that intervention cycle had no or at least one MD. Vertical line denotes the theoretical threshold EA of 30 kcal kg^−1^ FFM day^−1^ demonstrated by Loucks et al. [15]. Reproduced from Lieberman et al. [20]. **d** The predicted probability of experiencing an abnormal menstrual cycle increased to over 50% if energy availability decreased below 30 kcal/kg FFM/day. *EA* energy availability, *EXCON* exercising control group, *ED* energy deficit, *Int* intervention period, *FFM* fat free mass, *MD* menstrual disturbance

In our laboratory, we have attempted to advance our understanding of the threshold index by examining the validity of 30 kcal/kg FFM/day as a threshold of energy availability, below which menstrual disturbances are observed, and above which ovulatory menstrual cycles are maintained [20, 24]. Our efforts have included (i) cross-sectional studies of exercising women with varying menstrual cycle statuses [24], and (ii) randomized controlled trials examining the effects of exercise expenditure and energy restriction on the induction of menstrual disturbances during three consecutive menstrual cycles in previously ovulatory women [19, 20]. In our cross-sectional study [24] in 91 exercising women, we categorized menstrual status as either amenorrheic, oligomenorrheic, or eumenorrheic, and further subdivided the eumenorrheic women into subclinical menstrual groups as either ovulatory, inconsistent, or anovulatory. We did not find that an energy availability value of 30 kcal/kg FFM/day was able to differentiate subclinical menstrual disturbances; however, the threshold of 30 kcal/kg FFM/day did discriminate clinical menstrual status, i.e., amenorrhea was successfully discriminated from eumenorrhea [24]. As such, energy availability of 30 kcal/kg FFM/day may be useful in large groups of women to determine an at-risk profile. However, and most importantly, we were unable to substantiate and support the threshold of 30 kcal/kg FFM/day in our carefully designed randomized controlled trial of manipulating energy availability with both exercise and diet restriction. We randomized 35 sedentary, ovulatory women to a diet and exercise intervention of varying degrees of negative energy balance (Fig. 2a) over three menstrual cycles, following two control ovulatory menstrual cycles [19, 20]. We monitored menstrual status by assessing daily urinary estrone-1-glucuronide and pregnanediol glucuronide, and mid-cycle LH in daily urine samples collected throughout the intervention and baseline control time-periods [19]. Menstrual disturbances were observed throughout the intervention (Fig. 2b), but no specific value of energy availability emerged as a threshold below which menstrual disturbances were induced [20]. In fact, many disturbances were induced above the threshold of 30 kcal/kg FFM/day, and alternatively, menstrual disturbances failed to be induced in some women who were below the threshold of 30 kcal/kg FFM/day (Fig. 2c) [20]. Interestingly, we did observe that the incidence of menstrual disturbances increased in a linear manner as energy availability decreased [20]. Another point of importance was that the average age of our subjects was 20.3 ± 0.3 years, well below the age (29 years) at which Loucks et al. have shown that women fail to respond to low energy availability metabolically (insulin, IGF-1/IGF-1 binding protein ratio) or by effects on LH pulsatility [25].

Thus, our findings do not support the use of an absolute energy availability threshold of 30 kcal/kg FFM/day as a strategy to prevent the occurrence of abnormal menstrual cycles and other Triad sequelae. Rather, our findings highlight the presence of individual variability in the level of energy availability at which susceptibility to menstrual disruption is observed, and calls for more research and a rethinking of how best to utilize measures of energy availability in the exercising woman. Based on our results, we propose that a dose–response continuum exists between energy availability and menstrual abnormalities, such that when energy availability/energy deficiency decreases, the likelihood of a menstrual abnormality increases [19, 20]. This is supported by our findings that the predicted probability of experiencing an abnormal menstrual cycle increased to over 50% if energy availability decreased below 30 kcal/kg FFM/day (Fig. 2d) [19, 20]. We further propose that energy availability should be used in conjunction with other measurements of metabolic status and eating behaviors to assess an at-risk profile for Triad sequelae. Measurements that can be serially monitored in the same free-living individual, such as body weight and composition, resting metabolic rate, and measures of restrictive or under-eating, may be more informative and predictive of the initial suppression of reproductive function than adopting a particular absolute value of energy availability that was derived from short-term laboratory-based studies. Furthermore, in situations when only a single assessment is possible and indirect calorimetry is available, calculating a measured-to-predicted RMR ratio (mRMR/pRMR) may be useful for identifying metabolic suppression and an at-risk profile associated with Triad sequelae [26–30]. Our laboratory has previously utilized a mRMR/pRMR ratio of < 0.90 as a surrogate indicator of energy deficiency/low energy availability [30, 31], and we have recently tested the accuracy of the proposed 0.90 cut-off value to detect low TT_3_ indicative of energy deficiency [26]. Further, since multiple prediction equations are available for use, which will result in different ratio calculations, we tested the performance of the Harris–Benedict [32], Cunningham [33], and DXA [34] prediction equations to determine whether ratios derived from all calculation methods performed similarly. Overall, we found that ratios calculated from each of the prediction equations significantly predicted low TT_3_, but that a 0.90 cut-off should not be applied universally for all equations. Rather, a higher ratio should be utilized when using DXA-derived ratios, for which we propose a value of 0.94 [26].

We do agree that a sliding scale value of energy availability around a range targeting 45 kcal/kg FFM/day likely reflects a healthy level of energy availability for exercising women and we support the goal of developing good and reliable strategies to identify profiles that are at-risk for Triad sequelae in order to help prevent and treat the Female Athlete Triad. Since significant new evidence has been available since 2007, we suggest a revision to position stands and consensus statements regarding the use of energy availability in exercising women and in the Female Athlete Triad.

## Energy Availability Associated with an Initial Working Model of a Triad-like Syndrome in Male Athletes

### The Model

In the first publication that identified the Female Athlete Triad in 1992 [1], it was also mentioned that some attention should be given to male athletes to determine if a similar model resulting from poor energetic status was also apparent in male athletes. In 2019, the Female Athlete Triad Coalition renamed the organization the Female and Male Athlete Triad Coalition to reflect that a similar syndrome of energetic, reproductive, and bone health problems is occurring in men, and is to be formally recognized as the Male Athlete Triad.

### Low Energy Availability in the Etiology of the Triad: A Focus on Eating Behaviors

Similar to women, the most clinically worrisome symptoms of low energy availability in male athletes and exercising men are its effects on the hypothalamic–pituitary–gonadal (HPG) axis and on bone health. However, it must also be recognized that in men, similar to women, low energy availability may have an etiology related to poor eating behaviors [35, 36], to include disordered eating or clinically significant eating disorders.

In general, athletes display higher rates of eating disorders compared to the general population, and eating disorders are most commonly observed in athletes who participate in sports that favor a lean body type [35]. Notably, however, across all sport types the prevalence of male athletes identified as at-risk for an eating disorder and those with diagnosed eating disorders are lower than that observed in female athletes, 9% versus 21% and 8% versus 20%, respectively [35]. This pattern is supported by findings in male and female distance runners, where one report found that 46% of women were identified as at-risk for an eating disorder compared to only 14% of men [37]. It is interesting, however, that, although the prevalence of eating disorders in male athletes is lower than that of female athletes, it is similar to the prevalence of eating disorders in the general female population, suggesting that eating behaviors/eating disorders are an important concern in exercising men [35].

It is possible that the reported prevalence of eating disorders/disordered eating in men is underestimated, since most instruments used to identify disordered eating behaviors and restricted eating patterns were developed for use in women. Moreover, since societal and cultural body ideals differ between men and women, it is likely that disordered eating behaviors may differ as well [38]. As such, it may not be appropriate to use the same tools/subscales to identify disordered eating in men (to determine those at greatest risk for a male Triad-like syndrome) as those used in Female Athlete Triad research. Indeed, an emphasis must be placed on identifying and developing sex-specific risk factors and assessment tools for men. The disordered eating behaviors most commonly studied in exercising women at risk for the Triad include high cognitive dietary restraint [29, 39, 40], as measured by the Three-Factor Eating Questionnaire [41], and high drive for thinness [28, 30], as measured by the Eating Disorders Inventory [42], both of which have often been used as surrogate measures of energy deficiency and as screening tools to identify women at-risk for Triad-related conditions. However, there is far less information currently available on how cognitive restraint and drive for thinness relate to disordered eating and energy deficiency in exercising men. There are currently few reports of cognitive restraint in male athletes, none of which are specific to a population of exercising men. Drive for thinness, however, has often been applied to both sexes, and, generally, men tend to have lower drive for thinness subscale scores than women [43, 44]. For example, in a clinical population of men and women receiving treatment for an eating disorder, both sexes presented with elevated drive for thinness scores compared to a healthy population [43], but men presented with a lower drive for thinness scores compared to women (14.44 versus 20.32) [44]. In men and women who did not have clinical eating disorders, the mean drive for thinness scores are again lower in men than women and were reported as 5.6 versus 9.9 for those in the USA, and 2.3 versus 6.6 for an international sample, respectively [43]. Because men consistently report lower drive for thinness subscale scores than women, sex-specific cut-off values indicative of subclinical restricted eating patterns and underlying energy deficiency must be developed rather than relying on previously used definitions of high drive for thinness (≥ 7), which were developed from exercising women [28, 30].

Alternatively, entirely different subscales or tools may be necessary for the identification of disordered eating behaviors in exercising men. One such tool is the Drive for Muscularity Scale, which represents one’s perception that they are not muscular enough and which was developed and validated by McCreary and Sasse [45]. Drive for muscularity may be a valuable screening tool for identifying men at risk of developing eating disorders or disordered eating behaviors, since 27.5% of adolescent men and 4.9% of young women reported trying to gain weight or build muscle, with 21.9% of men and 4.5% of women exhibiting disordered eating behavior [46]. However, the associated disordered behaviors may not necessarily be associated with restrictive eating patterns or contribute to low energy availability since many of those who reported disordered eating behaviors also reported that they ate more or differently in a manner to gain weight or build muscle [46].

Interestingly, there may be an interaction between thinness and muscularity in the development of disordered eating—what has been referred to as a “drive for leanness” and which may be relevant to a Triad-like condition in men [47]. The mesomorphic body type, a combination of muscular and thin, may be the most relevant in the development of disordered eating in men [48], and drive for leanness refers to an interest in having relatively low body fat and toned muscles [47]. Both muscularity and thinness were independently and positively associated with elevated disordered eating in college-aged men as assessed by the Eating Disorder Examination Questionnaire [48]. One such drive-for-leanness scale was developed and significantly correlated with both drive for thinness and drive for muscularity in the entire sample, as well as in men and women independently. When looking for tools or risk factors that may be applicable to both sexes, drive for leanness may be useful particularly because there were no observed differences between men and women in drive for leanness, whereas women presented with higher drive for thinness scores and men displayed higher drive for muscularity [47]. Additional work is required, however, to relate drive for leanness with specific restrictive eating patterns.

In summary, much additional work must be conducted to better understand disordered eating behaviors in men, especially those that are related to restrictive eating patterns and therefore likely contribute to the development of low energy availability. Similar to women, the men at greatest risk for having disordered eating and eating disorders are those who participate in leanness sports [35], and tools aimed at identifying subclinical disordered eating must be developed for application in the population of exercising men.

### Low Energy Availability in the Etiology of the Triad: A Focus on Reproduction

Cross-sectional reports of hypogonadotropic hypogonadism have been reported in male athletes, particularly in those athletes participating in endurance sports, and include evidence of low testosterone [49–52], poor semen quality/oligospermia [53, 54], and low libido [55, 56]. As of 2019, only two investigators have tried to evaluate the effects of modulating exercise and dietary intake to vary the levels of energy availability from low to adequate in men [22, 23] as has been done in women [15]. Koehler et al. [23] manipulated energy availability to a level of 15 kcal/kg FFM/day versus 40 kcal/kg FFM/day in exercise trained males aged 25.2 ± 1.0 years. At an energy availability of 15 kcal/kg FFM/day, reductions in serum leptin and insulin were observed, but no reductions in serum TT_3_, IGF-1, or testosterone were observed irrespective of whether the low energy availability conditions were achieved by restricting dietary intake or by increasing energy expenditure [23]. Papageorgiou et al. [22] conducted a somewhat similar study in men and reported that at an energy availability of 15 kcal/kg FFM/day no reductions in serum TT_3_, insulin, leptin, or IGF-1 were observed. These findings are in stark contrast to those observed in the Loucks et al. [15, 16] studies in women where TT_3_, insulin, leptin, and IGF-1 were all suppressed at mild to modest levels of low energy availability ranging from 10 to 30 kcal/kg FFM/day.

Interestingly, it is primarily in “extreme” situations consisting of high intensity, long duration exercise or simultaneous exposure to multiple stressors that significant reductions in metabolic and reproductive hormones are observed. For example, during Army Ranger training where men were exposed to sleep deprivation and psychosocial stress, in addition to the primary stressor of energy deficiency, significant reductions in metabolic and reproductive hormones were observed [57]. Specifically, 8 weeks of training resulted in significant energetic changes including declines in total body mass, fat mass, and FFM, reductions in the concentrations of TT_3_, IGF-1, and insulin, and increases in the concentrations of cortisol and GH. Additionally, reproductive suppression was also observed, including decreased concentrations of testosterone, which fell below the normative range of values, as well as a reduction in LH concentration in a subgroup of men who underwent more severe caloric restriction [57]. There are also several examples of single bouts of prolonged, strenuous, outdoor exercise, such as ~ 160–1200 km running and cycling races, which induced hypogonadal states marked by reduced testosterone [58–60] and LH [59] concentrations, as well as metabolic hormone alterations including suppression of leptin and IGF-1 [58] and increases in cortisol [59, 60] and GH [60].

In cross-sectional studies of chronic strenuous exercise training, it seems that very high training loads are required for impairments to be translated to the HPG axis, presumably through poor energetic status. We found that high mileage runners (108.0 ± 4.5 km/week), compared to moderate distance runners (54.2 ± 3.7 km/week) and controls, had lower testosterone levels as well as poor semen quality, including decreased sperm motility, an increased immature sperm number, and decreased bovine cervical mucus penetration, all of which are associated with infertility [53]. It is important to note that the moderate mileage runners in our study maintained a gonadal and semen profile that was similar to that of the sedentary control group, despite running approximately 40–60 km/week. We concluded that in male athletes participating in high-volume training, the findings of decreased testosterone and abnormal semen profiles (Table 1) likely reflect the failure of these athletes to increase energy intake in a manner that accommodates the increased energy expenditure associated with a high training volume [53, 54].

**Table 1:** Comparison of semen characteristics in male runners. Adapted from De Souza and Miller [54], with permission

Studies	Runners		Control group	*p* value
	High-mileage (> 100 km/week)	Moderate-mileage (< 100 km/week)		
Arce et al. [83]				
Sperm density (× 10^6^/mL)	78 ± 12		176 ± 25	0.003
Forward progression (%)	40.8 ± 4.7		58.7 ± 2.4	0.005
Nonmotile (%)	54.2 ± 4.9		39.3 ± 1.9	0.005
Normal sperm (%)	40.2 ± 2.1		47.0 ± 3.3	< 0.05
Immature sperm (%)	17.2 ± 2.4		10.9 ± 1.2	0.035
Round cells (× 10^6^)	8.3 ± 1.7		2.5 ± 0.9	0.001
Sperm penetration (mm)	22 ± 5		43 ± 7	0.036
Bagatell and Bremmer [84]				
Sperm count (× 10^6^/mL)		119.9 ± 64.4	108.9 ± 91.7	NS
Total sperm/ejaculate (× 10^6^)		436.8 ± 64.6	316.1 ± 79.8	NS
Oval forms (%)		81.1 ± 1.8	78.9 ± 2.7	NS
Motility (%)		82.0 ± 4.6	73.2 ± 3.5	NS
Griffith et al.^a^ [85]				
Sperm count (× 10^6^)		108 ± 56	77 ± 61	NS
Jensen et al.^b^ [86]				
Semen volume (mL)	2.7 ± 1.4	2.8 ± 1.4		NS
Count (× 10^6^/mL)	133 ± 141	71 ± 65		0.001
Motility (%)	54 ± 9	55 ± 8		NS
Morphology (%)	15 ± 6	11 ± 7		0.001
De Souza et al. [53]				
Sperm density (× 10^6^/mL)	88.5 ± 14.8^c^	127.2 ± 32.2	175.5 ± 24.9	0.045
Normal motile count (× 10^6^)	58.5 ± 10.8^d^	118.8 ± 20.3	106.7 ± 22.3	0.052
Motile count (× 10^6^)	134.5 ± 23.9^c^	240.1 ± 45.3	224.7 ± 39.1	0.037
Forward progression (%)	40.3 ± 4.3^c^	48.8 ± 4.5^e^	58.7 ± 2.4	0.006
Nonprogressive (%)	6.1 ± 1.4^f^	7.3 ± 1.1^f^	2.0 ± 1.0	0.014
Nonmotile (%)	53.6 ± 4.4^c^	43.9 ± 3.9	39.3 ± 1.9	0.023
Immature sperm (%)	16.8 ± 2.2^d^	10.1 ± 2.0	10.9 ± 1.2	0.031
Round cells (× 10^6^)	8.0 ± 1.6^d^	2.5 ± 1.1	2.5 ± 0.9	0.004
Sperm penetration (mm)	26.8 ± 6.3^c^	37.5 ± 7.2	43.2 ± 7.0	0.024

*NS* no significant difference

^a^Prospective design

^b^High-mileage 60–160 km/week, low-mileage ≤ 55 km/week

^c^High-mileage runners vs. sedentary control group

^d^High-mileage runners vs. moderate-mileage runners and control group

^e^Moderate-mileage runners vs. control group

^f^High-mileage runners and moderate-mileage runners vs. control group

Notably, although highly trained men often have testosterone concentrations that are lower compared to untrained men, they often still fall within the normal physiological range, and these findings are consistent across a range of training volumes [49, 50, 52]. Runners averaging > 64 km/week had lower free and total testosterone concentrations compared to sedentary controls, with only one of the 31 runners (3%) falling below the normal range [50]. Runners averaging ~ 80 km/week were also within the normal range, albeit at the lower end [49], and a group of trained men averaging > 450 min of exercise per week of predominately endurance activities had lower concentrations of total and free testosterone compared to sedentary controls, but were again within the normal range [52].

Commensurate with alterations in testosterone and semen profiles, LH pulse frequency is significantly decreased in highly trained male runners (125–200 km/week) when compared to untrained controls [51]. In runners with a lower training load (~ 80 km/week), no difference in LH pulse frequency was observed compared to non-runner controls [49, 61], although one study reported that runners had lower LH pulse amplitude and area under the curve [49]. Similarly, no differences in LH pulse characteristics were observed between endurance-trained men averaging > 450 min/week of exercise compared to sedentary controls, including LH pulse frequency and amplitude [52].

Although many of the reported reproductive findings are from cross-sectional studies that observed reproductive suppression coincident with high training volumes, there is also evidence from experimental models of caloric restriction alone, which reduced LH pulse frequency in both men and male monkeys [62, 63]. We propose that poor energetic status is underlying the suppression of the HPG axis, and those who are participating in particularly high volumes of training may not be increasing their energy intake in a sufficient manner to match the increased energy expenditure associated with their training volume. It is likely that very high training loads present an energetic challenge, what we referred to as a “volume-threshold effect” over 20 years ago [53, 54]. It also appears to be difficult to consume the energy required to overcome such an energetic challenge, which, presumably, results in energy deficiency, which when extreme and chronic, translates into outcomes affecting the HPG axis.

Due to the absence of an overt clinical sign, such as a change in menstrual cycle frequency, reproductive suppression will likely be more difficult to assess in men than in women. But interestingly, male sexual function has been related to testosterone concentration [64] and changes in libido may be a helpful cue when trying to identify reproductive suppression secondary to energy deficiency in exercising men. For example, trained men reported higher scores on the Aging Male Symptoms questionnaire compared to controls [65], and a lower training volume has been associated with an increased likelihood of having high/normal libido based on a modified questionnaire comprising questions from the Aging Male Symptoms, Androgen Deficiency in the Aging Male, and Sexual Desire Inventory questionnaires [55].

Of note, the metabolic and reproductive perturbations observed in men also appear to recover more quickly following a reduction in exercise or increased caloric intake. Within 1 month of completing Army Ranger training all metabolic and reproductive hormones returned to normal levels, with most hormones recovering in a single week, and testosterone appeared to be highly responsive to refeeding [57]. In fact, following the aforementioned acute endurance events, testosterone concentrations rebounded towards baseline levels within 12 h of race completion [58], and were fully recovered within 2–3 days [59, 66]. One case study has been reported of an adolescent male athlete who presented with hypogonadotropic hypogonadism associated with energy deficiency through a combination of excessive exercise and undernutrition and in whom testosterone concentration was normalized within the 1-year follow-up period following reduced training volume and increased caloric intake [56]. Lastly, in non-human primates, refeeding after a single day of fasting resulted in an increase in both LH pulsatility and testosterone concentration, such that hormonal recovery progressively improved as the size of the refeed meal increased, thereby supporting that reversal of HPG axis suppression following a period of energy deficiency is likely due to an increase in hypothalamic central drive [67].

In summary from the data published to date, it seems apparent that the energetic and reproductive presentation of the Triad in men is different from that in women, whereby women are impacted more aggressively than men by low energy availability and that men are more resistant to the effects of low energy availability [15, 22, 23]. The reproductive axis of women also appears to recover more slowly than the rapid recovery observed for the reproductive axis in men [56–58, 66, 67].

### Low Energy Availability in the Etiology of the Triad: An Overview of Impaired Bone Health in Exercising Men

Most of the data in support of impaired bone health in male athletes are the product of cross-sectional investigations in leanness sports. Overall, longitudinal data or controlled and intervention-based investigations on the effect of energy restriction on bone mineral density (BMD) in male athletes are scarce. Similarly, reports on bone strength, geometry, and structure in exercising men with low BMD and bone stress injuries are not yet available. However, there is evidence that particular subsets of male athletes, such as male distance runners, who are at high risk for having low energy availability are also reported to have low BMD scores [8, 68] and a high prevalence of bone stress injuries [7, 69]. Additionally, there is evidence that bone turnover markers are influenced by periods of energy restriction [22, 70].

Low BMD scores have consistently been reported in men who participate in leanness sports, i.e., distance runners, cyclists, and jockeys [71–75]. For example, of 42 adolescent runners, 21% had *Z* scores ≤ − 1.0 at the lumbar spine and 5% had Z-scores ≤ − 2.0 [68]. In adolescent runners, 24% had a BMD Z-score < -1.0 and 4% had a BMD *Z* score of ≤ − 2.0; this is in contrast to only 6% and 0% of non-runner athletes having *Z* scores < − 1.0 and < − 2.0, respectively [8]. Similarly, one study reports that 32% of competitive cyclists had *Z* scores of ≤ − 2.0 [75], while 29% of a sample of 79 male flat jockeys and 13% of a sample of 69 male jump jockeys had spine BMD *Z* scores ≤ − 2.0 [76]. Although most investigations of bone health in male athletes did not assess energetic status, endurance and weight class athletes have been reported to have low energy availability/energy deficiency. Notably, energy availability has been reported as low as 18.8 ± 12.1 kcal/kg lean body mass (LBM)/day in competitive male cyclists (*n* = 6, age 29–49 years) with low BMD (*Z* scores ≤ − 1.0) [77], and as low as 19 kcal/kg LBM/day in jockeys [78]. Lastly, risk factors for low BMD in exercising men have been identified and include low body weight (< 85% expected), average weekly running mileage > 30, and previous stress fracture [8].

In addition to low BMD scores, male athletes are also reported to have a high incidence of bone stress injuries [7, 69]. Among 80 collegiate runners, 27% sustained at least one bone stress injury over a prospective 2-year period [7]. Further, bone stress injuries were predicted by a modified version of the Female Athlete Triad Cumulative Risk Assessment tool that was adapted in a manner to be applicable to male athletes and included low energy availability, low body mass index (BMI), prior bone stress injury, and low BMD as risk factors [7]. Additional positive risk factors identified in adolescent male runners include previous fracture and participation in a greater number of competitive seasons [69].

With respect to bone turnover markers, restricting energy by ~ 50% for 3 days decreased bone formation in trained runners [70]. Alternatively, 5 days of restricted energy availability (15 kcal/kg FFM/day) did not affect markers of bone turnover (N-terminal propeptide of type I collagen and C-terminal telopeptide type I collagen) in men [22]. The non-significant finding in men, compared to a significant 13% reduction in bone formation and 19% increase in bone resorption in women, suggests that, similar to the metabolic and reproductive systems, the effects of low energy availability on bone turnover in men are different to those in women whereby women are impacted more aggressively than men in response to low energy availability [22].

## Summary, Conclusion, and Future Directions

Based on the evidence to date, we propose a revision to the current thinking regarding the effects of energy availability in female and male athletes. On a broader level, we propose a rethinking of the use of energy availability in the assessment of energetic status in exercising women and men, and we do not support the use of an energy availability threshold in exercising women to indicate a point of susceptibility to menstrual disorders. We suggest that other assessments of energetic status must be considered, evaluated, and used to track athlete health and to predict an at-risk Triad profile.

In men, there is an interesting body of evidence available to date that points to a Triad-like syndrome in male athletes, although additional experimental studies are necessary to fully understand the effects of low energy availability/energy deficiency in exercising men. The strongest support, to date, for a Triad-like syndrome in exercising men is (i) a higher prevalence of eating disorders and disordered eating behaviors in male athletes compared to controls, particularly in those who participate in lean sports [35], (ii) reproductive suppression indicated by decreased testosterone concentration and/or reduced LH pulsatility following periods of energy deficiency [57, 62, 63] and reversal upon refeeding or increased caloric intake [57, 67], (iii) the association of high training volumes with low libido [55, 65], poor semen quality [53, 54], and low testosterone [49, 50, 52], and (iv) reports of low BMD [8, 68, 75, 76] and increased risk of BSI [7, 69] in athletes who participate in sports that have a lean component and in which low energy availability has been reported [77, 78]. While there are some observational, cross-sectional, and a few prospective experiments, much work in the form of scientifically rigorous studies remains to elucidate the energetic nuances involved in metabolic and reproductive suppression and poor bone health in male athletes. From the data available to date, it appears that male athletes have a resilient HPG axis, and perturbations that are observed return to baseline very quickly [57, 58, 66, 67], which is very different to the slow return to baseline observed in women [21, 79]. Much remains unknown about energy availability in male athletes, and certainly important manipulations of energy and exercise to test varying levels of energy availability in large cohorts of men are needed to understand energetic status in men beyond our current state of knowledge.

Recently, the concept of Relative Energy Deficiency in Sport (RED-S) has been proposed as a replacement to the Female Athlete Triad and has since been the topic of several papers [80, 81]. While RED-S has brought new interest to investigations regarding low energy availability and energy deficiency in men, it is largely a rebranding of Triad science and it has always been acknowledged by Triad researchers that a condition similar to the Female Athlete Triad likely exists in men [1, 2, 5, 82]. As a Male Athlete Triad model is established, methods similar in scientific rigor to those used to develop its female counterpart should be replicated such that quality of evidence, causality between components, and clinical relevance are considered and highlighted. An analysis of the features of each model and an evaluation of the scientific quality of the evidence underlying the Triad and RED-S have been published recently [3]. Further, there are likely to be sex-specific differences regarding the effects of energy availability on reproduction and bone, and it is not merely sufficient to apply evidence derived from female athletes directly to male athletes. High-quality investigations need to be conducted specifically in exercising men as well as comparisons between sexes in order to continue to improve our understanding of the effects of low energy in men and women participating in exercise and sport.
